# Single-tube isothermal label-free fluorescent sensor for pathogen detection based on genetic signatures

**DOI:** 10.3389/fchem.2022.951279

**Published:** 2022-09-01

**Authors:** Mark A. Reed, Yulia V. Gerasimova

**Affiliations:** Department of Chemistry, University of Central Florida, Orlando, FL, United States

**Keywords:** pathogen detection, signal amplification, isothermal molecular assay, transcription–based assay, light-up aptamers, three-way junction (3WJ) probe

## Abstract

We report on a single-tube biosensor for real-time detection of bacterial pathogens with multiplex capabilities. The biosensor consists of two DNA probes, which bind to the complementary fragment of a bacterial RNA to form a three-way junction (3WJ) nucleic acid structure. One of the probes encodes a fluorescent light-up RNA aptamer under T7 promoter. It allows for generation of multiple aptamer copies due to elongation and transcription of the 3WJ structure in the presence of the complementary target. The aptamer coordinates and thereby enhances fluorescence of a cognate fluorogenic dye, allowing for fluorescent detection of the RNA target. Multiple aptamer copies can be produced from a single target-dependent 3WJ structure allowing for amplification and visual observation of the signal. The limit of detection depended on the assay time and was found to be 1.7 nM or 0.6 nM for 30-min or 60-min assay, respectively, when *N*-methylmesoporphyrin IX (NMM) was used as a fluorescent indicator. The sensor is excellent in analyzing folded RNA targets and differentiating between closely related sequences due to the multicomponent character of the target-interrogating probe. Response to unamplified samples of total bacterial RNA from *Mycobacterium tuberculosis* complex or *Escherichia coli* was observed with excellent selectivity within 30 min under isothermal conditions at 50°C in a one-tube one-step assay. Several bacterial species can be detected in multiplex by utilizing biosensors with the template strands encoding different light-up aptamers. The isothermal one-tube-one-step format of the assay and the possibility to monitor the signal visually makes it amenable to use in a point-of-care scenario.

## Introduction

Detection of bacterial pathogens is of significance for disease diagnostics and surveillance, as well for monitoring food and water samples (Lazcka et al., 2007). This importance stimulates a constant quest to improve the existing detection techniques and to develop the new ones. Some newly explored avenues take advantage of nanotechnology (Fu et al., 2020; Li et al., 2021) and instrumental advances (Bi et al., 2020; Wang et al., 2021), among others. But the golden standard for species-specific bacterial identification remains to be the analysis of pathogen-specific genetic signatures using molecular diagnostic assays. Most of the current commercially available tests for molecular diagnostics of infectious diseases are based on polymerase chain reaction (PCR), which requires laboratory settings, highly trained personnel, and expensive instrumentation (Louie et al., 2000). Recently, isothermal amplification of nucleic acid fragments (Li and Macdonald, 2015) has been explored to improve affordability of nucleic acid amplification tests (NAATs). Unprecedented sensitivity of nucleic acid detection attainable by NAATs has a downside—even a tiny drop of a previously produces amplicon carried over to new samples can cause false-positive results (Fredricks and Relman, 2000).

An alternative approach to NAATs is to amplify a signal rather than the nucleic acid target, for example, with the help of enzymes (Gerasimova and Kolpashchikov 2014). Some of the signal-amplification strategies are paired with junction probes (also known as multicomponent, or binary, probes) (Wang and Tao, 2020), which offer advantages of target differentiation down to a point-mutation resolution (Kolpashchikov 2010), and interrogation of highly structured nucleic acids, such as rRNA or tRNA (Reed et al., 2020). One type of the junction probes is based on the formation of the three-way junction (3WJ) structure, which is made of three duplex domains forming a junction point (Wu et al., 2004). The 3WJ probes have been previously suggested to allow for the enzyme-based target-dependent release of signaling sequences (Wharam et al., 2001; Wharam et al., 2007; Murakami et al., 2012; Fujita et al., 2016; Guo et al., 2020; Lee et al., 2020; Kim et al., 2021; Zhang et al., 2021). One of the earliest reported transcription-based signal amplification strategies utilizing a 3WJ probe was the signal-mediated amplification of RNA technology (SMART) (Wharam et al., 2001). It utilized a downstream enzyme-linked oligosorbent assay (ELOSA) for the detection of the released signaling sequences. The need of this downstream assay for signal detection in addition to a two- or three-step protocol, which required several hours of incubation at different temperatures, hindered practical applications of the technology.

In the present study, with these shortcomings in mind, we have developed a one-tube-one-step isothermal and real-time assay based on the target-induced 3WJ formation, its extension and transcription of the extended template to produce multiple copies of a light-up RNA aptamer, thus enabling real-time signal monitoring. Light-up aptamers have been previously suggested as label-free signal transducers in assays detecting ions (DasGupta et al., 2015; Li et al., 2015; Chen et al., 2018; Raducanu et al., 2020), proteins (Bang et al., 2012) or enzymatic activities (Wu et al., 2015; Svensen and Jaffrey, 2016), miRNAs (Wang et al., 2019; Park et al., 2022), as well as viable bacterial cells (Zhang et al., 2020). In this proof-of-principle work, we used malachite green aptamer (MGA) (Grate and Wilson 1999), as well as a guanine quadruplex (G4)-folded structure, which is known to enhance fluorescence of *N*-methylmesoporphyrin IX (NMM) (Ren and Chaires 1999), as fluorescent reporters. The reporters were encoded in the 3WJ structure to be generated *via* transcription only in the presence of a 16S rRNA target. 16S rRNA is a classical target for phylogenetic analysis and genotype-based classification of bacterial species (Yang et al., 2016). Ribosomal RNA constitutes about 85% of total bacterial RNA (Karpinets et al., 2006), which makes rRNA sequences attractive targets due to their abundance. Here, we demonstrated detection of RNA from two clinically significant bacterial species—*E. coli*, which is one of the predominant causative agents of the urinary tract infections (Lee et al., 2018), and *Mycobacterium tuberculosis* complex (MTC) comprising etiological agents of tuberculosis (Frieden et al., 2003). The assay successfully differentiated *M. tuberculosis* from non-tuberculous mycobacteria based on the difference in their 16S rRNA sequences. Moreover, both *E. coli* and *M. tuberculosis* RNA can be detected simultaneously in a multiplex fashion when two specific 3WJ systems that encode orthogonal RNA reporters are combined.

## Materials and methods

### Materials

Oligonucleotides were custom made by Integrated DNA Technologies, Inc. (Coralville, IA) and were used without purification. Non-DEPC treated DNase/RNase-free water was from Boston Bio Products (Ashland, MA). *Bsm* DNA polymerase, ultra-low range DNA ladder, RiboRuler High Range RNA ladder, Middlebrook 7H9 with OADC, Luria-Bertani (LB) broth, and Ambion Turbo DNA-free DNase kit were obtained from ThermoFisher Scientific (Waltham, MA). Hi-T7 RNA polymerase, Hi-T7 RNA polymerase buffer, ribonucleotide triphosphate (rNTP) mix, and deoxyribonucleotide (dNTP) mixes were purchased from New England BioLabs (Ipswich, MA, United States). GelRed^®^ nucleic acid gel stain was from Biotium (Fremont, CA, United States). *N-*methylmesoporphyrin IX (NMM) and malachite green were purchased from Sigma-Aldrich (St. Louis, MO, United States). Dithiothreitol (DTT) and ammonium chloride were purchased from Acros Organics (Fair Lawn, NJ, United States). All other reagents were purchased from Fischer Scientific.

### Isolation of total bacterial RNA

Total RNA from mycobacterial species was isolated following the previously published protocol (Rohde et al., 2007). In brief, cultures (50 ml) of *M. bovis* BCG, *M. simiae*, or *M. abscessus* were grown to log phase (OD_600_ = 0.5–0.8) in 75 cm^2^ tissue culture flasks in 7H9-OADC medium. Pelleted bacilli were lysed in 65°C Trizol using a BeadBeater and 0.1 mm silicon beads. Total RNA was isolated from Trizol lysates by chloroform extraction and Qiagen RNeasy column purification. DNA contamination was removed using an Ambion Turbo DNA-free DNAse kit according to manufacture protocol. Following DNAse treatment, the concentration of RNA was measured using a NanoDrop UV-Vis spectrophotometer (ThermoFisher Scientific, Waltham, MA, United States).

To isolate total RNA from *E. coli* (strain DH10B), the cells were grown an OD_600_ of 1 in LB medium, pelleted by centrifugation and processed using a Monarch Total RNA Purification kit from New England Biolabs (Ipswich, MA, United States) according to the vendor-recommended protocol. The isolated RNA was analyzed by 0.8% agarose gel electrophoresis. The concentration of 16S rRNA in the total RNA preparation was determined by comparing the intensity of the corresponding band in the agarose gel with the intensity of the band containing a 1500-nt marker of the RiboRuler High Range RNA ladder.

### Fluorescent assay

Samples (50 μL) containing EP (0.1 μM), TP (0.1 μM), 0.1 U/μL *Bsm* DNA polymerase, 2.5 U/μL Hi-T7 RNA polymerase, 0.125 mM dNTPs, 2.5 mM rNTPs, 1 × Hi-T7 RNA polymerase buffer, 5 mM NH_4_Cl, 5 mM DTT, 20 mM MgCl_2_, 2 μM NMM and/or malachite green (MG) were incubated in the absence or presence of synthetic DNA target (0–100 nM) at 50°C for 30 min or 60 min. The signal was measured using a Cary Eclipse Fluorescence Spectrophotometer (Agilent, Santa Clara, CA, United States) using a 3-mm cuvette. For NMM, the signal was measured at 608 nm upon excitation at 399 nm, with the excitation/emission slits of 5/10 nm. For MG, the signal was measured at 650 nm upon excitation at 617 nm, with the excitation/emission slits of 10/10 nm. In both cases, PMT was set to “medium” (600 V). The data was processed in Microsoft Excel and/or OriginLab^©^ 2018b graphing software. Tube images were acquired using a smartphone camera upon exciting the samples’ fluorescence using a Spectroline^™^ Slimline^™^ UV Transilluminator.

### Gel electrophoresis

Polyacrylamide gel (PAAG) electrophoresis under native conditions was performed using 10% PAAG supplemented with 10 mM MgCl_2_. The gels were run at a constant voltage of 80 V in 1 × TBE buffer at 50°C. Denaturing PAAG electrophoresis was performed using 12% PAAG supplemented with 7M urea in 1 × TBE buffer at 7 V/cm, 50°C. The gels were stained with 1 × GelRed DNA staining dye, and the gel images were acquired using a BioRad GelDoc XR + Molecular Imager coupled with ImageLab software.

### Calculation of the linear dynamic range and limit of detection (LOD)

Linear dynamic range was determined by plotting the fluorescent signal as a function of the target concentration and fitting the experimental data using the linear trendline of OriginLab^©^ 2018b graphing software. Linear dynamic range corresponds to the range of target concentrations, at which the signal is directly proportional to the concentration. The LOD was calculated using a 3 σ/S rule (MacDougall and Crummett, 1980), where σ is the standard deviation for the blank, and S is the slope of the linear trendline for the signal concentration dependence in the linear dynamic range. The data processing was done using OriginLab^©^ 2018b graphing software.

### Detection of 16S rRNA as a component of total bacterial RNA preparations

To verify that the assay can be used for the detection of a native RNA sample, total bacterial RNA from either *E. coli* or mycobacteria was used as a target to detect a fragement of 16S rRNA interrogated by the correspondent 3WJ system. Samples (50 μL) containing EP (0.1 μM), TP (0.1 μM), 0.1 U/μL *Bsm* DNA polymerase, 2.5 U/μL Hi-T7 RNA polymerase, 0.125 mM dNTPs, 2.5 mM rNTPs, 1 × Hi-T7 RNA polymerase buffer, 5 mM NH_4_Cl, 5 mM DTT, 20 mM MgCl_2_, and 2 μM NMM were incubated in the absence or presence of total bacterial RNA (17 or 30 ng/μL) at 50°C for 30 min. The signal was measured at 608 nm upon excitation at 399 nm using a Cary Eclipse Fluorescence Spectrophotometer (Agilent, Santa Clara, CA, United States) with a 3-mm cuvette. The data from three independent trials was processed in Microsoft Excel.

## Results

### Mechanism of signal amplification

**FIGURE 1:**
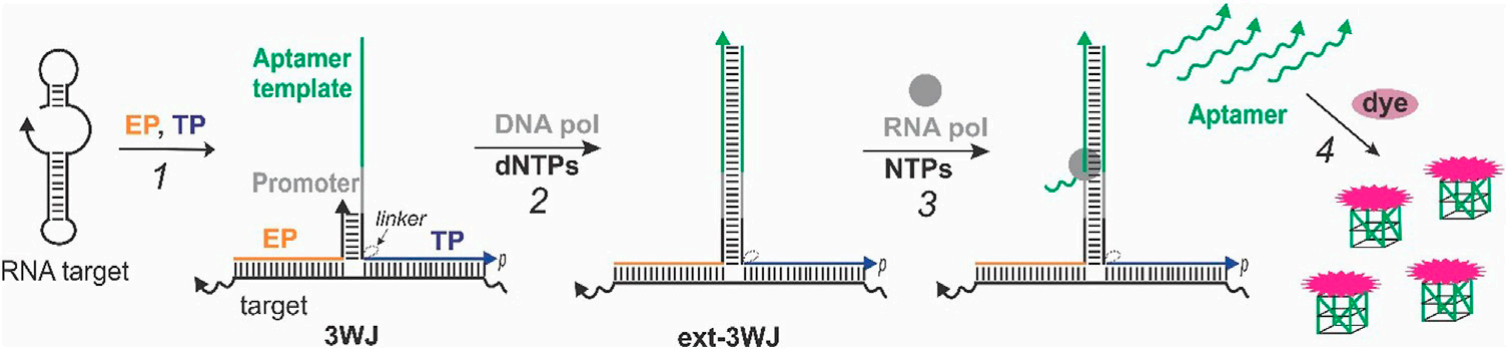
Real-time light-up aptamer-expressing 3WJ system. The signal is due to the following steps occurring in the same sample: (1) An RNA target is interrogated by the template probe (TP) and extension probe (EP) to form a 3WJ structure; (2) the 3’-end of EP in the 3WJ structure is extended to form T7 RNA polymerase promoter sequence and the template encoding the sequence of a light-up aptamer, which is complementary to the “aptamer template” fragment of TP; (3) the RNA polymerase recognizes the promotor sequence and generates multiple copies of the encoded RNA aptamer, which acquires the active dye-binding conformation to enhance fluorescence of a cognate fluorogenic dye (4).

The aptamer-expressing probe assay relies on the formation of a 3WJ structure consisting of a nucleic acid target of interest and two strands—template probe (TP) and extension probe (EP) (Figure 1, step 1). The TP sequence has a fragment complementary to the T7 RNA polymerase promoter adjacent to a fragment complementary to a light-up dye-binding RNA aptamer (“aptamer template”). The 3′-terminal EP fragment is complementary to the TP sequence next to the promoter region. This enables formation of a short duplex between EP and TP to enable elongation of the free 3′-end of EP by *Bsm* DNA polymerase into an extended 3WJ structure (ext-3WJ) (Figure 1, step 2). To prevent elongation of TP by the DNA polymerase, its 3′-end is phosphorylated. The ext-3WJ structure contains the T7 RNA polymerase promotor sequence and a “gene” encoding for the RNA aptamer. Hence, T7 RNA polymerase synthesizes multiple copies of the aptamer using ext-3WJ as a template (Figure 1, step 3). We used a thermostable T7 RNA polymerase genetically engineered to have optimal activity at 50–52°C, which allows to perform the assay at 50°C for improved target interrogation and shorter reaction time.

Each aptamer molecule folds into an active dye-binding conformation and binds its cognate dye, thereby enhancing the dye’s fluorescence (Figure 1, step 4). As a result, each molecule of the nucleic acid target triggers multiple signaling events reporting the target’s presence. It should be noted that the aptamer sequence is absent unless the target triggers its enzymatic synthesis. This feature allows using the system in a one-step one-pot format and monitoring the signal in real time. We encoded one of the two RNA aptamer sequences - MGA that lights up malachite green (Grate and Wilson 1999) and a G4-folded structure, which is known to bind and enhance fluorescence of NMM (Ren and Chaires 1999). The G4-expressing system is advantageous due to the reported high stability of RNA G4 structures (Zhang et al., 2011), which allows the usage of the system at 50°C with thermostable enzymes to destabilize secondary/tertiary structures of the target and reduce the assay’s time.

Due to the sharing of the target-interrogating function between two probes—EP and TP, the system allows for excellent selectivity in target recognition. To enable synthesis of the aptamer sequence, which serves as a signal transducer, both strands need to bind to their complementary target fragments adjacent to each other. This would not be possible if the target contains one or more mismatched nucleotides in the fragment designed to interact with one or both strands. For excellent selectivity, the length of the mismatched probe-target duplex needs to be minimized (Demidov and Frank-Kamenetskii, 2004), which would compromise the probe-target affinity for monolith but not multicomponent probes (Kolpashchikov 2010). In multicomponent probes, one of the target-interacting components can be short and therefore responsible for the probe’s selectivity, while another can be long and responsible for high affinity of the probe to the target. The longer component of the probe also performs a target-unwinding function, which is especially important for natural RNA targets intrinsically folded into stable secondary/tertiary structures (Noller and Woese, 1981). Such nucleic acid targets are challenging for conventional monolith probes (Lima et al., 1992). In this proof-of-principle work, we designed the EP and TP probes targeting a fragment of 16S rRNA from *E. coli* (NCBI accession number NC_010473.1) or from *M. tuberculosis* (NCBI accession number NR_102810.2). Ribosomal RNAs are known to exhibit stable secondary and tertiary interactions that would challenge efficiency of conventional hybridization probes (Herschlag et al., 2018). The use of the split approach helped to mitigate this challenge.

### Aptamer-expressing 3WJ systems targeting 16S rRNA from *Mycobacterium tuberculosis* complex

**FIGURE 2:**
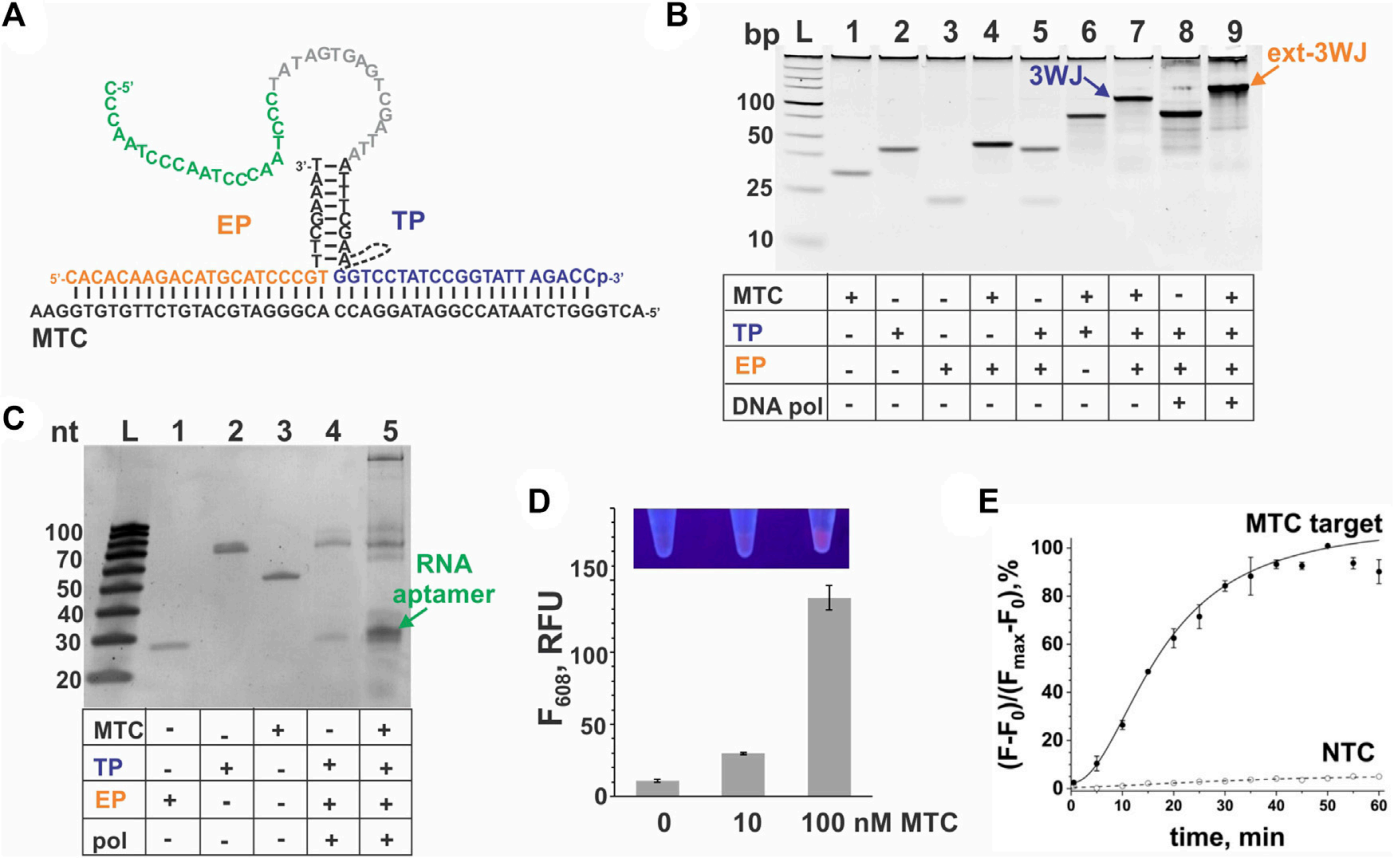
3WJ system expressing the NMM-binding G4 sequence in response to MTC. **(A)**. 3WJ complex formed between EP and TP strands in the presence of the MTC target. The dashed line connecting two fragments of the TP strand represents the hexaethylene glycol (*heg*) linker. The nucleotides corresponding to the promoter complement and the aptamer-encoding domain are in grey and green, respectively. **(B)**. Effect of a non-nucleotide linker on the fluorescent signal of the MTC-G4-3WJ system in the absence or presence of 100 nM MTC target. NTC stands for no-target control. The samples were incubated at 50°C for 30 min **(C)**. Analysis of the 3WJ complex and the product of its extension by *Bsm* DNA polymerase by native gel electrophoresis. All oligonucleotides were used at 100 nM. The samples were pre-incubated at 50°C for 30 min. **(D)**. Analysis of the G4 sequence expression in the presence of MTC (100 nM) by the system containing both *Bsm* DNA polymerase and T7 RNA polymerase using denaturing gel electrophoresis. **(E)** Fluorescent response of the system to MTC (10 or 100 nM). The bar corresponding to the no-target control is labeled as “0”. All samples were incubated at 50°C for 30 min. Inset: Visual observation of the signal in the sample upon excitation with a transilluminator. **(F)** Instantaneous monitoring of the signal triggered by 100 nM MTC target or background signal (NTC). For panels **(B)**, **(E)** and **(F)**, the data is average of three independent experiments, with error bars as standard deviations.

For interrogation of 16S rRNA from MTC, we designed a 3WJ system expressing a G4-forming RNA sequence. The sequences for the probes and a synthetic DNA mimic of 16S rRNA—MTC—used for the system’s optimization and characterization are listed in Table 1. The expected structure of the 3WJ complex formed between MTC-G4-TP*heg*, MTC-EP and the synthetic target MTC is shown in Figure 2A. It was observed that a hexaethylene glycol (*heg*) linker connecting the target-binding and the EP-binding fragments of TP helps to decrease the background signal (no-target control, NTC) without significantly affecting the target-induced NMM fluorescence (Figure 2B), which agrees with previously reported SMART assay performance (Wharam et al., 2001). Therefore, we used MTC-G4-TPheg in subsequent experiments. The slow-migrating complex corresponding to 3WJ is observed in gel under native conditions upon mixing the three components at equimolar concentrations (Figure 2C, lane 7). As expected, elongation of the complex by *Bsm* DNA polymerase further decreases the mobility of the resultant ext-3WJ structure (Figure 2C, lane 9). When both DNA polymerase and T7 RNA polymerase were added, a band migrating at around 30-nt ssDNA marker slightly above the EP probe was observed in the target-containing sample but not in the NTC sample (Figure 2D, lane 4) green arrow in Figure 2D, lane 5. This confirms synthesis of the encoded RNA aptamer in the target-containing sample. The aptamer generation results in the enhanced NMM fluorescence observed in the presence of the target (Figure 2E). The signal is observed due to folding of the generated RNA into a G4 structure, which was reported to bind and enhance fluorescence of environment sensitive dyes including NMM (Sabharwal et al., 2014). Remarkably, the fluorescent signal is bright enough to be easily visualized by a UV transilluminator (Figure 2E, inset). As expected for a signal amplification-based assay, the signal of the 3WJ system triggered by the cognate nucleic acid target accumulates over time and plateaus within 35–40 min, while the background signal does not change much (Figure 2F). Even though appreciable signal can be obtained within 5–10 min after the target addition, we have selected the 30 min as the assay time, which serves as a compromise between the assay time and the signal intensity.

**TABLE 1:** Oligonucleotides used in this study.

Name	Sequence*^a^*	ΔG, kcal/mol*^b^*
MTC-G4-TP	CCCAATCCCAATCCCAATCCCTATAGTGAGTCGTATTAATTTCGAA **GGTCCTATCCGGTATTAGACC**/3Phos/	0.00
MTC-G4-TP*heg*	CCCAATCCCAATCCCAATCCCTATAGTGAGTCGTATTAATTTCGAA/iSp18/**GGTCCTATCCGGTATTAGACC**/3Phos/	0.00
MTC-MGA-TP	GGATCCATTCGTTACCTGGCTCTCGCCAGTCGGGATCCTATAGTGAGTCGTATTAATTTCGAA **GGTCCTATCCGGTATTAGA**/3Phos/	−3.14
MTC-EP	**CAC​AAG​ACA​TGC​ATC​CCG​T** TT​CGA​AAT	0.00
MTC	ACT​G**GG​TCT​AAT​ACC​GGA​TAG​GAC​CAC​GGG​ATG​CAT​GTC​TTG​TG**G​TGG​AA	−3.42
EC-G4-TP*heg*	CCCAATCCCAATCCCAATCCCTATAGTGAGTCGTATTAATTTCGAA/iSp18/**CATCTGGGCACATCCGATGGC**/3Phos/	0.00
EC-EP	**CCC​ACC​TAC​TAG​CTA​ATC​C** TT​CGA​AAT	0.00
EC	GCC​TCT​T**GC​CAT​CGG​ATG​TGC​CCA​GAT​GGG​ATT​AGC​TAG​TAG​GTG​GG**G​TAA​CGG​CTC​ACC	−3.63

a /iSp18/and/3Phos/are hexaethylene glycol (*heg*) linkers and 3′-terminal phosphate groups, according to the IDT nomenclature. Complementary fragments of the oligonucleotides are in bold or underlined. T7 RNA polymerase promotor sequence is in grey.

b Calculated for NUPACK predicted secondary structures at 0.06 M Na^+^, 44 mM Mg^2+^, and 50°C, to simulate assay conditions. NUPACK does not have the capability to estimate the free-energy contribution of non-nucleotide linkers or alternative structures (e.g., guanine quadruplexes), so the calculated free energy values for the *heg*-containing oligonucleotides are the same as for their linker-less counterparts.

**FIGURE 3:**
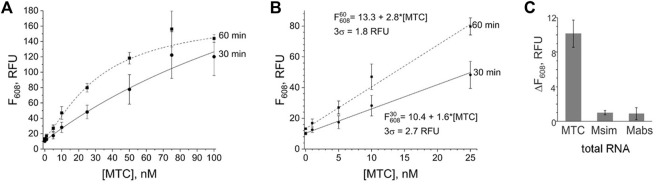
Limit of detection and selectivity of the G4-MTC 3WJ system. **(A)** Dependence of the fluorescence intensity at 608 nm on MTC concentration upon incubation of the samples at 50°C for 30 min or 60 min. **(B)** Limit of detection for 30-min or 60-min assay. **(C)** Selectivity of the system in response to total bacterial RNA (17 ng/μL) from *M. bovis* BCG (MTC), *M. simiae* (Msim) or *M. abscessus* (Mabs). The signal is expressed as the fluorescence of the target-containing sample after subtraction of the no-target control. The data is averaged from three independent experiments, with standard deviations as error bars.

**FIGURE 4:**
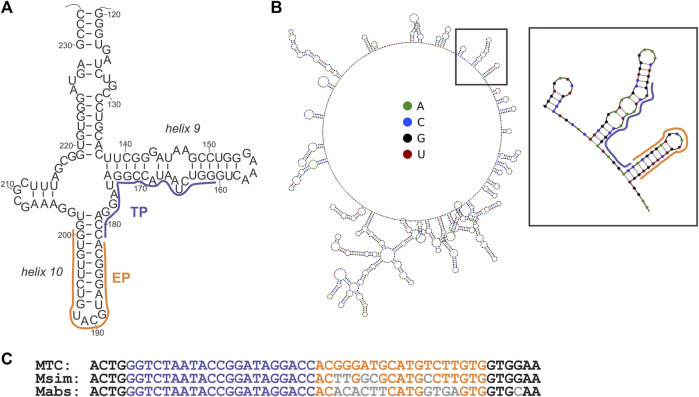
Structure of the MTC target. **(A)** The secondary structure of a fragment of 16S rRNA from *M. tuberculosis* (Kempsell et al., 1992) interrogated by the target-binding fragments of EP and TP of the cognate 3WJ system, as indicated by orange and purple lines, respectively. **(B)** Minimal energy secondary structure of 16S rRNA (sequence from accession number NR_102810.2) as predicted by Nupack software (Zadeh et al., 2011) at 50°C, with the probe-interrogated fragment indicated by a black square and enlarged. **(C)** Alignment of the targeted fragment of the 16S rRNA gene from the indicated mycobacterial species. Nucleotides complementary to the target-binding fragments of EP and TP strands are in orange and purple, respectively. Nucleotides of *M. simiae* (Msim, NCBI accession number NR_026081.1) or *M. abscessus* (Mabs, NCBI accession number NR_042917.1) differing from the same positions in the gene from MTC are in grey. Structure of the MTC target.

The signal of the MTC-G4 3WJ system increases with the target concentration in the range of 0–75 nM at both 30-min and 60-min assay time (Figure 3A). The linear dynamic range depended on the assay time and was determined to be 0–75 nM and 0–25 nM for 30 and 60 min, respectively (data not shown). The limit of detection (LOD) calculated using the 3σ-rule and 0–25 nM linear dynamic range is 1.7 nM and 0.6 nM for the 30-min and 60-min assay, respectively (Figure 3B). These LOD values, while unable to compete with the LODs of PCR-based assays (Braun et al., 2021), are in the same range as the LOD reported for the molecular beacon probe (MBP) (Kolpashchikov 2012). Unlike MBP, which cannot efficiently interrogate a structured nucleic acid target (Nguyen et al., 2011; Reed et al., 2020), the split junction probe used in the aptamer-expressing assay can tolerate stable secondary/tertiary structures a natural RNA target generally acquires. Here, this advantageous property of the split probes was used to target native 16S rRNA sequences of bacterial species. Specifically, the EP and TP strands of the G4-MTC 3WJ system were designed to interrogate helices 9 and 10 of MTC 16S rRNA (Kempsell et al., 1992) (Figures 4A,B). Indeed, total RNA from *M. bovis* (17 ng/μL) containing ∼10 nM 16S rRNA (assuming 16S rRNA constitutes ∼30% total bacterial RNA) triggered the signal of a similar intensity as in the presence of 10 nM synthetic MTC target (Figure 3C, compare with Figure 2E). Response of the MTC-G4 3WJ system to total RNA from two non-tuberculous mycobacterial species—*M. simiae* (Msim) and *M. abscessus* (Mabs)—did not exceed the fluorescence intensity corresponding to LOD (Figure 3C). The fragments of the 16S rRNA genes from the mycobacterial species interrogated by TP are identical, and only the duplexes formed by the target and EP have several mismatches (Figure 4C). This makes it feasible to utilize one and the same TP strand for identification of different mycobacterial species, if combined with a species-specific EP, which is an inexpensive unmodified deoxyribonucleotide.

**FIGURE 5:**
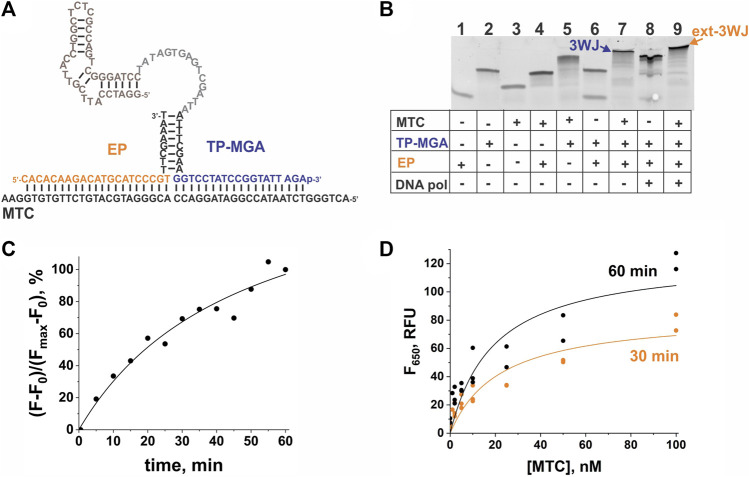
Performance of the 3WJ system expressing the malachite green aptamer (MGA) sequence in response to MTC. **(A)** 3WJ complex formed between EP and TP strands in the presence of the MTC target. The nucleotides corresponding to the promoter complement and the aptamer-encoding domain are in grey and brown, respectively. **(B)** Analysis of the 3WJ complex and the product of its extension by *Bsm* DNA polymerase (ext-3WJ) using native polyacrylamide gel electrophoresis. All oligonucleotides were used at 100 nM. The samples were pre-incubated at 50°C for 30 min. **(C)** Instantaneous monitoring of the fluorescent signal at 650 nm upon excitation at 617 nm triggered by 100 nM MTC. The data is expressed in the percent turn-on calculated by taking the maximal fluorescence as 100% after subtraction of the background signal. **(D)** Dependence of the fluorescence intensity at 650 nm on MTC concentration upon incubation of the samples at 50°C for 30 min or 60 min. The data from three independent experiments were plotted together and fitted with a hyperbolic function using Origin 2018.

To demonstrate a modular character of the aptamer-expressing 3WJ system, we have designed and tested another MTC-interrogating TP—MTC-MGA-TP (Table 1), which encodes MGA instead of the G4 sequence. The MTC-MGA 3WJ system performed similarly to the MTC-G4 system, with slightly different kinetics and concentration dependence (Figure 5). These discrepancies may reflect the difference in length of the synthesized RNA aptamers (21 nts and 38 nts in G4 and MGA, respectively) and in their affinity to the cognate fluorogenic dyes (Babendure et al., 2003; Sabharwal et al., 2014; Perenon et al., 2020).

### Multiplex target detection

**FIGURE 6:**
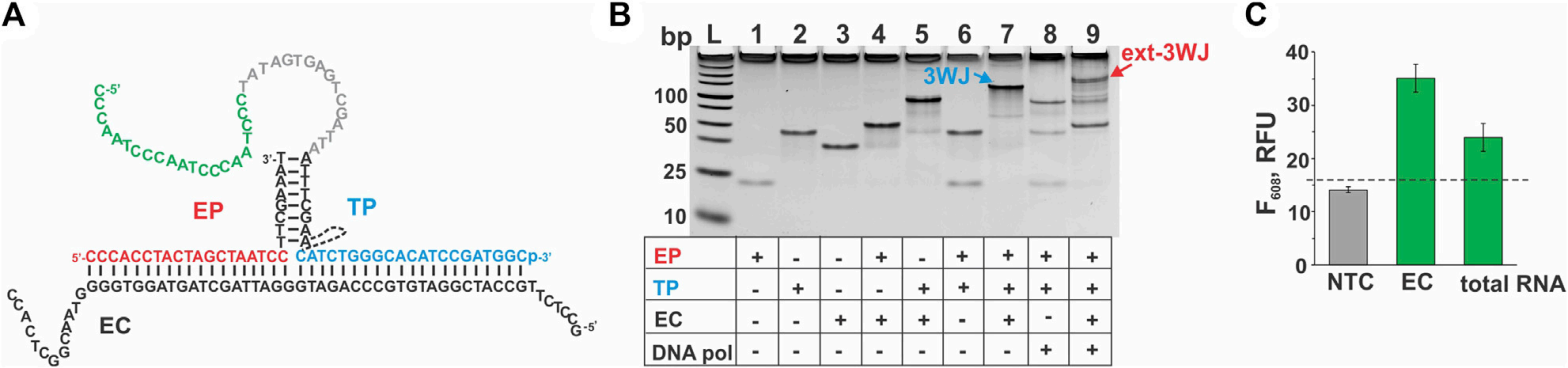
Performance of the 3WJ system expressing the G4-folding sequence in response to a fragment of *E. coli* 16S rRNA. **(A)** 3WJ complex that presumably forms between EP and TP strands in the presence of the EC target. The dashed line connecting two fragments of the TP strand represents the *heg* linker. The nucleotides corresponding to the promoter complement and the aptamer-encoding domain are in grey and green, respectively. **(B)** Analysis of the 3WJ complex and the product of its extension by *Bsm* DNA polymerase (ext-3WJ) by native polyacrylamide gel electrophoresis. All oligonucleotides were used at 100 nM. The samples were pre-incubated at 50°C for 30 min. **(C)** Fluorescent response of the system to the absence of targets (NTC, “no-target control”) or to the presence of either synthetic target EC (20 nM) or total *E. coli* RNA preparation containing 30 ng/ul of 16S rRNA. The samples were incubated at 50°C for 30 min. The data is averaged from three independent experiments, with error bars as standard deviations. The dashed line shows the fluorescent intensity threshold corresponding to the average intensity of the NTC sample plus 3 standard deviations. The signal above the threshold indicates the presence of the interrogated target.

**FIGURE 7:**
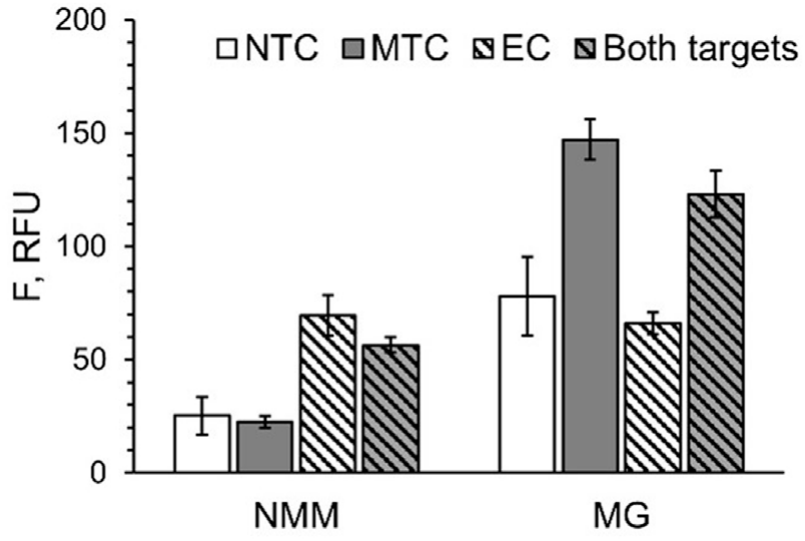
Multiplex detection of MTC and EC targets with G4- and MGA-expressing 3WJ systems. All samples contained EP and TP strands from both systems in the absence of the targets (NTC) or in the presence of either MTC, EC or both targets simultaneously. Fluorescence of all samples was measured under conditions for NMM emission (at 608 nm upon excitation at 399 nm) and MG emission (at 650 nm upon excitation at 617 nm). The data is averaged from three independent experiments, with standard deviations as error bars.

In some applications, it may be important to detect several pathogens simultaneously. Therefore, we combined two EP/TP sets—MTC-MGA-TP/MTC-EP yielding MGA in response to MTC, and EC-G4-TP*heg*/EC-EP (Table 1), which responds to a fragment of *E. coli* 16S rRNA by G4 expression. Presumable binding of the *E. coli*-specific system to the synthetic DNA mimic of the target (EC) to form a 3WJ complex, as well as gel electrophoretic analysis of the complex formation and fluorescent response of the system to EC or total *E. coli* RNA, are shown in Figure 6. When both MGA- and G4-expressing systems were mixed, high emission of NMM fluorescence was observed only when EC target was present, and the presence of MTC target was needed to observe high emission of MG fluorescence (Figure 7). This proof-of-principle experiment demonstrates feasibility of the multiplex aptamer-expressing 3WJ probe assay. More detailed evaluation of the analytical performance of the assay in the multiplex mode necessitates optimization of the reagent concentrations and/or sequences of the encoded aptamers.

## Discussion

The assay reported here combines such advantages of split probes as high selectivity and ability to interrogate highly structured nucleic acid targets with the convenience of label-free signalling sequence of an RNA light-up aptamer generated in multiple copies in response to the target’s presence. The selectivity of the system can be fine-tuned by varying the length of the target-recognizing fragments of EP and/or TP. Here, the selectivity function was pertained to only the EP strand, which allowed differentiation between *M. tuberculosis* complex and non-tuberculous mycobacteria. In this case, the affinity of the 3WJ probe to the target was maintained by the length of the TP strand. Previously, we have shown that even if one component of the split probe is shortened to allow for discrimination of point mutations, the length of the target-recognizing fragment of another probe’s component controls the overall affinity to keep the signal high (Gerasimova et al., 2015; Reed et al., 2020). This illustrates the advantage of split hybridization probes over monolith ones—the ability to cope with the affinity-specificity dilemma that may compromise performance of monolith probes (Demidov and Frank-Kamenetskii, 2004).

The assay exhibited the LOD in the nanomolar range. This LOD value is limited by the requirement of having a high enough concentration of the generated RNA light-up aptamer to trigger the signal. Indeed, the dissociation constant for MGA complex with malachite green was reported to be 117 nM, with the appreciable quantum yield observed at 0.1–1 μM aptamer concentration (Babendure et al., 2003). Affinity of G4 structures to NMM was also characterized with *K*_d_s in a submicromolar range (Sabharwal et al., 2014). These values necessitate formation of submicromolar concentrations of the encoded RNA reporters for the signal to be observed. *In vitro* transcription allows synthesis of up to 500 copies of the encoded RNA sequence per DNA template molecule (Pokrovskaya and Gurevich, 1994). Hence, 1 nM extended 3WJ structure would generate 500 nM aptamer at best. This would require at least nanomolar target concentration due to the stoichiometric interaction of the target to the EP and TP strands, which is also a limiting factor in case of conventional hybridization probes such as MBP (Kolpashchikov 2012). The use of light-up aptamers with higher affinity to their fluorogens could allow improvement of the detection limit. Moreover, the LOD for transcription-based assays can be improved by extending the assay time, through optimization of reagent ratios for the transcription reaction (Becker and Masquida, 2011) or by the use of a more efficient promotor (Conrad et al., 2020).

In the aptamer-encoding 3WJ assay reported here, generation of the signalling sequence of an RNA light-up aptamer depends on the action of two enzymes—*Bsm* DNA polymerase for extension of the 3WJ structure formed with the RNA target to produce a double-stranded T7 promotor, and the T7 RNA polymerase for transcription of the encoded light-up aptamer. As much as the use of protein enzymes should be limited for an affordable molecular assay in the reported technology, thermostable enzymes for both 3WJ extension and transcription are employed. This increases stability of the assay components. Additionally, it allows the assay to be run at higher temperature (50°C) than a conventional 37°C transcription optimum. Elevated temperature contributes to greater accessibility of the target for probe’s binding.

It is possible to limit the use of enzymes for the light-up aptamer-encoding 3WJ technology to only RNA polymerase. When this paper was in preparation, a similar system was reported, in which DNA polymerase to produce the promotor sequence was avoided due to the use of a split T7 promoter (Yoon et al., 2022). In this case, the antisense promoter sequence was included in the same probe that also encoded a light-up aptamer (analogue of the TP component herein). The sense promoter sequence, which would otherwise be generated due to the 3WJ extension, was in two segments—one was part of the EP analogue and another hybridized to the target-mediated 3WJ structure from solution. Such design, however, may require an additional optimization of the promoter splitting site as re-formation of the double-stranded promoter sequence could be dependent on the stability of the 3WJ structure, which is target-dependent. In addition, Yoon and co-authors have demonstrated successful interrogation of multiple target’s sites. Such a strategy can be explored to improve the LOD for the assay reported here.

## Conclusion

We have developed a one-tube-one-step real-time label-free signal amplification molecular assay, which can detect the presence of bacterial rRNA isothermally within as little as 30 min in a mixture with other RNA sequences as part of total bacterial RNA. The target’s presence is monitored as increase in the fluorescence emission of a fluorogenic dye bound to a corresponded light-up RNA aptamer encoded by a strand of the 3WJ probe utilized by the assay. Modular design of the probe allows easy adaptation of the assay to a new pathogen, and for closely related species it is possible to re-use a more expensive TP strand and only tailor the unmodified EP strand to be species-specific. Several pathogens in one and the same sample can be simultaneously detected in a multiplex format if the target-specific probes express different aptamer sequences that light-up fluorogens with emission maxima at different wavelengths. The assay can be employed for the real-time detection of bacterial or viral pathogens.

## Data Availability

The raw data supporting the conclusions of this article will be made available by the authors, without undue reservation.
